# Social Support Mediates the Effect of Burnout on Health in Health Care Professionals

**DOI:** 10.3389/fpsyg.2020.623587

**Published:** 2021-01-13

**Authors:** Pablo Ruisoto, Marina R. Ramírez, Pedro A. García, Belén Paladines-Costa, Silvia L. Vaca, Vicente J. Clemente-Suárez

**Affiliations:** ^1^Department of Health Sciences, University of Navarre, Pamplona, Spain; ^2^Department of Psychology, Universidad Técnica Particular de Loja, Loja, Ecuador; ^3^Department of Statistics and Operations Research, University of Granada, Granada, Spain; ^4^Faculty of Sport Sciences, European University of Madrid, Villaviciosa de Odón, Spain; ^5^Research Group in Culture, Education and Society, Coast University, Barranquilla, Colombia

**Keywords:** burnout – professional, social support, psychological stress, general health, health care professionals

## Abstract

Burnout is characterized by emotional exhaustion and caused by exposure to excessive and prolonged stress related to job conditions. Moreover, burnout is highly prevalent among health care professionals. The aim of this study is, first, to examine the mediating role of social support over the effect of burnout in health care professionals and, second, to explore potential gender differences. A convenience sample of 1,035 health professionals from Ecuador, including 608 physicians and 427 nurses (68% women, with and age *M* = 40 + 9 years old), was surveyed using the *Maslach Burnout Inventory* (MBI), Social Support Survey (MOS), and *General Health Questionnaire* (GHQ-28) as measures of burnout, social support, and general health, respectively. Social support was found to mediate the negative effects of burnout on health regardless of gender. Differences across the three dimensions of burnout and health are further discussed, along with their implications for designing effective burnout interventions for health care professionals in Ecuador.

## Introduction

Burnout is considered the result of chronic stress in one’s work environment, meaning that the burnout process can be understood in terms of the stress-stress-coping framework proposed by Lazarus and Folkman (1984). Burnout was first described by Freudenberger (1974) based on a lack of energy among workers in direct contact with people. Years later, Maslach and Jackson (1986) distinguished three different dimensions of burnout in the work environment: emotional exhaustion, cynicism or depersonalization, and a diminished sense of personal accomplishment (Maslach, 2003; Maslach and Leiter, 2008). Emotional exhaustion corresponds with the notion of strain, which is linked to tension, anxiety, physical fatigue, and insomnia (Lee and Ashforth, 1990). Cynicism or depersonalization corresponds to the notion of coping or defensive behavior used to prevent (evitation) or reduce the stressor (scape) by treating others as objects or numbers, rather than people (Lee and Ashforth, 1990). A diminished sense of accomplishment is regarded as an outcome of the stress–strain coping sequence based on helplessness, while personal accomplishment is associated with proactive control as a coping mechanism (Lee and Ashforth, 1990).

Emotional exhaustion has been considered the most important dimension in burnout (Leiter and Maslach, 2009), although cynicism and lack of personal accomplishment have also been associated with an increased risk of turn over and job dissatisfaction (Brenninkmeijer and Van Yperen, 2003). In mid-2019, burnout syndrome was included by the World Health Organization in the 11th Revision of the International Classification of Diseases (ICD-11) as an occupational phenomenon (not classified as a medical condition).

Although burnout may occur in any job context, it is most notable among health care professionals where, for example, burnout can impact as many as half of all physicians (Gandi et al., 2011; Ebling and Carlotto, 2012; Koutsimani et al., 2019; Dall’Ora et al., 2020). This high prevalence is relevant because approximately 50% of medical leaves are due to burnout (Demerouti et al., 2002). Moreover, the current rates of burnout among health care professionals are considered a “ticking timebomb,” as increases in burnout incidence will result in deleterious effects on health (Hall et al., 2016; Dawkins and Burdess, 2020). Work overload, long work hours, time pressure, role conflicts, and insecurity seem to contribute to an increased risk of burnout (Amoafo et al., 2014; Muller et al., 2020; Pappa et al., 2020; Seo et al., 2020). Indeed, as burnout rates increase among health care professionals, anxiety and depressive symptoms also become more prevalent (Hintsa et al., 2016).

An ongoing discussion focuses on the relationship between burnout and depression. On the one hand, Bianchi et al. (2013) highlighted the overlap between burnout and depression for eight of the nine major depressive episode diagnostic criteria of the Diagnostic and Statistical Manual of Mental Disorders, considering burnout as “atypical” depression or depression “induced” by work (Bianchi et al., 2017a,b; Bianchi and Brisson, 2019). On the other hand, Koutsimani et al. (2019) defined burnout and depression as distinct constructs; thus, this paper should be included to reinforce the importance of studying burnout (Koutsimani et al., 2019). In both cases, burnout is currently a topic of special interest.

Another important discussion that has drawn attention in the scientific literature is the relationship between personality traits and burnout. Indeed, personality predispositions could explain burnout and changes in the level of burnout over time (Alarcon et al., 2009; Armon et al., 2012). In particular, neuroticism or a tendency to feel stress and a higher sensitivity to stress have been associated with an increased risk of emotional exhaustion, depersonalization, and diminished personal accomplishment, while emotional stability is associated with more active coping strategies and a lower risk of burnout (Alarcon et al., 2009; Swider and Zimmerman, 2010; Armon et al., 2012). In a study exploring sex differences in personality traits across 55 cultures, Schmitt et al. (2008) concluded that women reported higher levels of neuroticism than men.

Fortunately, a large number of studies have found that social support can ameliorate the negative effects of stress on health (Holt-Lunstad et al., 2010; Mikkola et al., 2018). As expected, social support has been associated with lower rates of burnout among health care professionals (Hou et al., 2020). Moreover, as health care professionals are, in fact, formal caregivers, a growing number of studies underline the important of establishing programs to give physicians social support as a core element (Holt-Lunstad et al., 2010; Applebaum, 2015). However, the state of the science on empirically supported interventions for health care professionals remains in its infancy (Applebaum, 2015) 5 years later. Our understanding of how social support protects health care professionals from the deleterious effects of burnout remains understudied and elusive, particularly in middle-income countries such as Ecuador.

The aim of this study was to analyze the mediating effects of social support on the three core dimensions of burnout and general health in health care professionals in Ecuador. To our knowledge, this is the largest study attempting to explore social support, burnout, and general health among health care professionals in Ecuador. This research will contribute to the development of interventions aimed at reducing the risk of burnout and improving health among health care professionals.

## Materials and Methods

### Participants

A convenience sample of 1,035 health care professionals, including 608 physicians and 427 nurses, was recruited for this study. All participants were selected from public and private health centers from seven cities in seven regions (out of 24 regions) of Ecuador. As inclusion criteria, all participants had a minimum experience of 4 years performing their duties. Participants failing to meet these criteria were excluded. All subjects gave their informed consent for inclusion before they participated in the study. The study was conducted in accordance with the Declaration of Helsinki and was approved by the local committee at the Public University of Loja (Ecuador) (code 05.1904.2017).

The final sample was made up of 1,035 health professionals, with 68.02% women (44.88% physicians, 55.11% nurses) and 32.08% men (88.22% physicians, 11.78% nurses). The participants were selected from seven regions of Ecuador: 21.8% from Loja (*n* = 226), 10.1% from Imbabura (*n* = 104), 10.2% from Azuay (*n* = 105), 17.4% from Guayas (*n* = 180), 31.4% from Pichincha (*n* = 325), 5.2% from Chimborazo (*n* = 54), and 3.9% from Pastaza (*n* = 40). The sample was comprised of those with a mixed ethnicity (88.21%) followed by Caucasians (9.66%), mostly from urban areas 91.48%. Ages ranged from 27 to 65 years old among the men (*M* = 42.62; Mdn = 41; SD = 10.16) and from 27 to 65 for the women (*M* = 40.29; Mdn = 39; SD = 9.02).

A total of 90% had full-time positions, with salaries ranging from around 1,000 to 6,000 USD for men (9.66% below 1,000; 43.50% between 1,000 and 2,000 USD; 37.76% between 2,000 and 3,000 USD; 1.8% between 4,000 and 5,000 USD; and 3.02% between 5,000 and 6,000 USD) and from around 1,000 to 4,000 for women (27.41% below 1,000 USD; 53.41% between 1,000 and 2,000 USD; 16.90% between 2,000 and 3,000 USD; and 1.56% between 3,000 and 4,000 USD). Almost half (48.64% of men and 59.80% of women) of the selected health care professionals worked with shifting schedules, mostly in the public sector (55.59% of men and 68.04% of women), followed by the private sector (18.42% of men and 21.16 of women). In addition, 25.98% of men and 10.79% of women worked in both the public and private sector.

Finally, no gender differences were found for working hours or workload, with an average of 9–10 h worked per day and almost 20 patients/day. In terms of experience, most participants reported around a decade of experience, with a slight differences of 1.5 extra years of experience (more in the men than the women). For a detailed description of the sociodemographic, see Table 1.

**TABLE 1:** Gender differences among the sociodemographic variables.

Sociodemographic	Males (*n* = 331) M ± SD	Females (*n* = 704) M ± SD	*t*	*p*	Cohen’s *d*
Age	42.6 **±** 10.2	40.2 ± 9.0	3.56	<0.01**	0.249
Work hours/day	10 **±** 4.3	9.5 ± 3.6	1.76	0.078	0.126
Number of patients/days	18.9 **±** 12.3	19.7 ± 13.6	−0.95	0.324	−0.061
Experience (years)	15.2 **±** 9.5	13.6 ± 8.2	2.69	0.01**	0.180

	**Males (*n* = 331) %**	**Females (*n* = 704) %**	**χ*^2^***	***p***	***Cramer’s V***

Marital status (S/M/D/W)	18.1/73.1/6/2.7	27.1/62.4/8/2.6	12.844	0.01**	0.111
Institution (public/private/public and private)	55.6/18.4/26	68/21.2/10.8	39.453	0.01**	0.195
Contract (temporary/permanent)	32.9/66.1	26.4/73.4	16.250	0.01**	0.125

Marital status: S, single; M, married; D, divorced; W, widowed.

**p < 0.01.

### Measures

All caregivers were interviewed in one session divided into two parts: a sociodemographic questionnaire [age, gender, marital status, institution (public or private), number of patients/day, contract (temporary/permanent)] and a psychological protocol using instruments developed and/or validated in Spanish. Specifically, the following standardized measures were included in this survey:

Maslach Burnout Inventory (MBI) (Maslach and Jackson, 1986; Spanish version from Seisdedos, 1997). The MBI consists of a self-administered 22 item scale used to measure burnout, which is based on the three different dimensions described in the introduction: emotional exhaustion (nine items), cynicism (five items), and a lack of professional efficacy (eight items). Participants responded on a 7-point scale (0 = never and 6 = daily). An example item was “*My job has too many physical demands*.” A burnout score was then calculated, weighing each dimension so that the scores corresponded to the original response scale (0.4 × exhaustion + 0.3 × cynicism + 0.3 × lack of professional efficacy), as suggested by previous studies (Ahola and Hakanen, 2007). The internal consistency for this study was good, with a Cronbach’s α of 0.72, for a total score of α = 0.86 for emotional exhaustion, α = 0.63 for depersonalization, and α = 0.75 for the lack of a professional subscale.

The Social Support Survey (MOS) (Sherbourne and Stewart, 1991; Spanish version, Revilla et al., 2005) consists of 20 self-administered items used to assess how often the subject can count on people to support them in different contexts. This scale provides an overall functional social support index and four functional support subscales: emotional, material, affective, and positive social interactions. Participants responded on a 5-point Likert-type scale ranging from “never” = 1 to “always” = 5. An example item is “*I can count on someone to share my worries with*.” The internal consistency for this study was high with a Cronbach’s α = 0.97.

The Personality Questionnaire Revised-Abbreviated (EPQR-A) (Francis et al., 1992; Spanish version, Sandín et al., 2002) consists of 24 items used to assess three personality traits: neuroticism, neuroticism, and a predisposition to feeling stress; extraversion or the tendency to seek the company of others and talk; and psychoticism or the tendency toward aggressiveness and interpersonal hostility. Neuroticism and extraversion are personality traits currently included in the “big five” higher-order personality traits. Scores range from 0 to 6 for each scale. An example item is “*I suffer significant changes in my mood*.” The internal consistency for this study was good, with a Cronbach’s α ranging from 0.63 to 0.78 (neuroticism α = 0.75, extraversion α = 0.77). The internal consistency for psychoticism was low (α = 0.23), so it was not included in the analysis.

The General Health Questionnaire (GHQ-28) (Goldberg and Hillier, 1979; Spanish version from Muñoz et al., 1979) consists of 28 items used to assess one’s current mental state (over the past few weeks). This scale provides four subscales: somatic symptoms, anxiety symptoms, social disfunction, and depressive symptoms. An example item is “*Are you always tired although you had a good night’s sleep?*” Participants respond using a four-point Likert scale (from 0 to 3). Scores ranged from 0 to 84. The internal consistency for this study was high, with a Cronbach’s α = 0.97.

### Design and Procedure

A cross-sectional correlational study was conducted. Data were collected from seven regions in Ecuador in late 2017, randomly selected out of 24 regions without using a probabilistic sampling method (see Supplementary Figure 1). All data were collected via a survey that included standardized sociodemographic scales, which was administered using printed material by a team of psychologists trained by the lead researcher. The durations of the sessions averaged 15 min.

### Data Analysis

All data analyses were performed using the Statistical Package for the Social Sciences, version 21, for Mac (IBM Spain, Madrid, Spain). The descriptive analysis of the data included the means and standard deviations (*M* + SD) for the quantitative variables, while frequencies and percentages were used for the nominal variables. Pearson’s tests and *t*-tests were used, respectively, to compute the correlations and to assess gender differences for the quantitative variables. Cohen’s *d* was used to test the effect size. Independent hierarchical multiple regression models were also applied to examine the effects of burnout (Step 1) and social support (Step 2) on health among health care professionals. The detection of multicollinearity was performed using the Variance Inflation Factor (VIF), with VIF > 5 as the cut-off point for the diagnosis of collinearity (Sheather, 2009). For multiple regressions, the R2 was obtained. Additionally, residual plots were used to assess the goodness of fit for the regression model. Finally, the indirect effects of social support on the effects of the three dimensions of burnout on health among health care professionals were examined using the bootstrap method via Process macro version 3.3 (Hayes, 2017) for SPSS and the interaction effects between gender. The number of bootstrap samples was set to 10,000. Baron and Kenny’s (1986) mediational triangle was also used to visually display the mediation effects. The general significance adopted was *p* ≤ 0.05.

## Results

### Gender Differences in Burnout and the Main Outcomes

First, the rates of burnout were significantly higher in men than women across the burnout measures, although the effect size was consistently small.

Second, no significant gender differences were found in somatic symptoms, anxiety symptoms, or depressive symptoms. Men reported significantly higher social disfunction, again with a very small sample size.

Third, no gender differences were found in social support regardless of the source of social support considered (emotional, material, social, or affective).

Finally, women reported significantly higher levels of neuroticism with a small effect size, while no significant differences were found in extraversion.

For a detailed description of the outcomes measured by gender, see Table 2.

**TABLE 2:** Gender differences in outcome variables.

Variables	Males (*n* = 331) M ± SD	Females (*n* = 704) M ± SD	*t*	*p*	Cohen’s *d*
Burnout (MBI)	63.9 ± 15.0	60.3 ± 14.6	3.66	0.001**	0.243
Exhaustion (MBI-E)	19.0 ± 11.6	17.9 ± 10.9	1.45	0.010**	0.197
Cynicism (MBI-D)	5.1 ± 5.4	4.1 ± 4.7	2.83	0.005**	0.191
Lack of personal accomplishment (MBI-A)	39.7 ± 7.9	38.1 ± 8.8	2.69	0.005**	0.197
General health (GHQ28)	43.0 ± 11.1	44.4 ± 11.1	−1.84	0.066	−0.126
Somatic symptoms (GHQ-SS)	0.9 ± 1.7	1.2 ± 1.8	−2.08	0.037*	−0.171
Anxiety symptoms (GHQ-A)	1.0 ± 1.8	1.1 ± 1.7	−0.15	0.878	−0.057
Depression symptoms (GHQ-D)	0.1 ± 0.8	0.2 ± 0.8	−0.33	0.739	−0.125
Social disfunction (GHQ-SD)	0.5 ± 1.0	0.4 ± 1.1	0.47	0.634	0.095
Social support (MOOS)	75.2 ± 19.7	75.0 ± 19.3	−0.19	0.845	0.010
Emotional (MOOS-E)	30.9 ± 8.8	31.0 ± 8.6	−0.19	0.845	−0.011
Material (MOOS-I)	15.5 ± 4.5	15.3 ± 4.5	0.41	0.676	0.044
Social (MOOS-S)	16.1 ± 4.2	16.0 ± 4.3	0.38	0.699	0.023
Affective (MOOS-A)	12.6 ± 3.1	12.6 ± 3.1	0.29	0.767	0.000
Neuroticism (EPQRA-N)	1.5 ± 1.6	1.7 ± 1.7	−2.24	0.025*	−0.121
Extraversion (EPQRA-E)	4.3 ± 1.8	4.1 ± 1.8	1.25	0.211	0.111

0.634*; **p < 0.01.

### Prediction of Health in Health Care Professionals

The hierarchical multiple regression showed that burnout significantly predicted health scores in GQH-28 (Step 1), accounting for 18.1% of the variability in the GQH-28 scores (*b* = 0.320, *p* < 0.001), and remained significant when social support was added (Step 2) (0.318, *p* < 0.001), significantly increasing the amount of variance of explained GQH-28 to 22% (Δ*F*_1_,_1032_ = 55.818, *p* < 0.001). Residual plots (*x* = the standardized regression predicted value, and y = the standardized regression residual) were randomly dispersed around the horizontal axis, supporting the appropriateness of the regression model.

### Mediation and Moderation Analysis of the Relationship Between Social Support, the Three Core Dimensions of Burnout and Health

**FIGURE 1:**
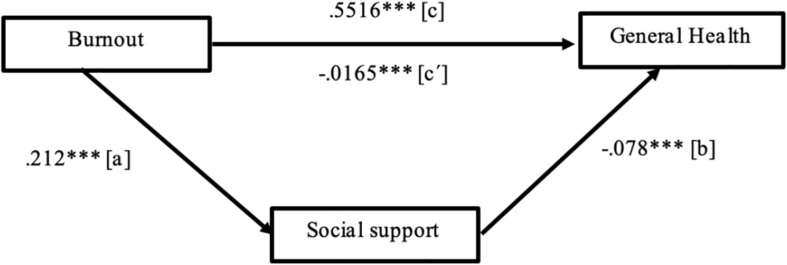
The unstandardized regression coefficients for the mediating effect of social support on the relationship between burnout and health. ***p < 0.0001.

First, social support mediated the effect of *exhaustion*, the core dimension of burnout syndrome, on health among health care professionals, accounting for 34.33% of the variance in health (*p* < 0.001). Exhaustion significantly predicted lower social support (path *a*), *b* = −0.212, *t*_(__1033__)_ = −3.952, *p* < 0.001. Social support significantly predicted lower health problems (path *b*), *b* = −0.078, *t*_(__1033__)_ = −5.351, *p* < 0.001. The direct effect of burnout exhaustion on health, when ignoring the mediator (path *c*), was significant, *b* = 0.5516, *t*_(__1033__)_ = 21.782, *p* < 0.001. Finally, the indirect effect of burnout exhaustion on health (path *c*′), after controlling for social support as a mediator, was also significant, *b* = −0.0165, *p* < 0.001, 95%, with a confidence interval (CI) ranging from −0.0069 to 0.0387. Furthermore, no differences were found in the indirect effect of social support between men and women (moderated mediation index = 0.004, SE = 0.0094, 95% CI ranging from −0.0144 to 0.0229). Following Baron and Kenny’s (1986) recommendations, Figure 1 visually displays the mediational triangle for the relationship between burnout exhaustion on health in health care professionals mediated by social support.

Second, social support also mediated the effect of *cynicism* (*b* = 0.0478, with a 95% IC: 0.0221 to −0.0797) and *lack of accomplishment* (*b* = −0.0466, with a 95% IC: −0.0705 to −0.0355) on health among health care professionals. However, the indirect effects of social support on burnout cynicism and a lack of accomplishment on health accounted for only 2.04 and 7.99% of the variance of health among health care professionals, respectively. These indirect effects of social support on burnout cynicism and a lack of accomplishment were also not moderated by gender, with a moderated mediation index of 0.0115, 95% IC (−0.0327 to 0.0560) and 0.0115, 95% IC (−0.0327 to 0.0560), respectively.

The complementary results showed that the indirect effect of social support on the effect of burnout exhaustion on health varied significantly across the different indicators of health. For example, the indirect effect of burnout on health mediated by social support accounted for 6.10% of the variance in depressive symptoms (c′ = 0.0011, SE = 0.0005, 95% IC = 0.0003; 0.0195), 25.72% of anxiety symptoms (c′ = 0.0016, SE = 0.0007, 95% IC = 0.0004; 0.0031), 25.02% of somatic symptoms (c′ = 0.0011, SE = 0.0006, 95% IC = 0.0001; 0.0025), and 19.53% of social disfunction symptoms (c′ = 0.0009, SE = 0.0005, 95% IC = 0.0002; 0.0020).

## Discussion

The aim of this study was to examine the mediator role of social support on the effect of burnout upon the general health of health care professionals—physicians and nurses—in Ecuador. This study is important because, to our knowledge, it is the largest survey assessing burnout, social support, and general health among health care professional in Ecuador.

The results of this study highlight the beneficial role of social support as a mediator between the negative effects of burnout on general health among health care professionals in Ecuador, which was unexplored prior to this study. These results are consistent with previous studies, which reported high rates of burnout among health care professionals in other countries (Li et al., 2018; Korkmaz et al., 2020; Zaed et al., 2020; Zaghini et al., 2020).

Burnout rates among health care professionals in Ecuador were lower than the rates previously reported among critical care health care professionals in other samples (Embriaco et al., 2007). Surprisingly, the burnout rates were higher among men than women, which contrasts with the original studies from Maslach and Jackson (1981). Moreover, female health professionals reported significantly higher levels of neuroticism, which previous studies have linked with worse health outcomes (Fornés-Vives et al., 2019; Kelly et al., 2020). This result is important because it suggests that even if neuroticism is related to burnout as a personality trait consistent with a predisposition to psychological stress, both may be distinct constructs. Interestingly, no gender differences were found in general health or social support. These results contrast with previous studies, which reported gender differences among health care professionals (Leiter and Maslach, 2009; Sweile, 2020).

The dimension of burnout known as emotional exhaustion was the single best predictor of general health in health care professionals. Indeed, emotional exhaustion accounted for more than one third of the variance in general health—three times the amount of variance accounted for by the other two dimensions of burnout (cynicism and a lack of personal accomplishment). Furthermore, emotional exhaustion predicted general health better than the total score of burnout alone. This is consistent with previous studies that considered emotional exhaustion as the core dimension of burnout (Pavlakis et al., 2010; Abraham and Jacobowitz, 2020).

Social support mediated the negative effects of burnout on the general health of both male and female health professionals. This was a robust effect found for the three dimensions of burnout (even though emotional exhaustion was the core dimension in terms of its impact on general health variability). In particular, more than 25% of the variability in anxiety symptomatology and social disfunction was accounted for by the reported burnout level. More studies are needed to fully understand how social support protects health care professionals’ health (Panagioti et al., 2018). However, based on the results of this study, randomized controlled trials that aim to reduce burnout, like the recent study conducted by Medisauskaite and Kamau (2019), are promising and should include evidence-based social support interventions.

However, the state of the science of empirically supported interventions for health care professionals remains in its infancy (Applebaum, 2015), as is our understanding of how social support protects health care professionals from the deleterious effects of burnout on health.

Notably, the healthcare professionals in the sample group were overrepresented by men and nurses. Moreover, a gender salary gap was found when considering the reported salary distribution, where women were overrepresented in the lowest salaries (below 1,000 USD) with a ratio of men/woman of 3:1, while men were overrepresented among the highest salaries (above 4,000 USD), with 5% of men, and no women, in this range. This result is consistent with previous literature that reported the feminization of most healthcare workplaces (Shannon et al., 2019) and the importance of continuing to break the current glass ceiling in health care for women. One reason for this salary gap might be that men tend to combine public and private practices more often than women or due to the potential assumptions of women having more domestic responsibilities. Furthermore, the evidence of dual practices in the health sector (public and private) in Ecuador is a widespread phenomenon around the world, which has its own implications for the equity, efficiency, and quality of health care provision (García-Prado and Gonzalez, 2007).

Finally, the results of this study must be taken with caution since this study is based on a cross-sectional correlational design with a convenience sample; therefore, this study has limitations in terms of its generalization to other populations or using it to infer causality. Future studies should further explore the mediating role of social support on the effects of burnout upon health among health care professionals using probabilistic samples with longitudinal designs and across a wider range of health indicators.

## Conclusion

First, male health care professionals in Ecuador reported significantly higher levels of burnout than females, while female health care professionals reported higher levels of neuroticism than males.

Second, social support ameliorated the negative effects of burnout on health among health care professionals in Ecuador regardless of gender. The burnout dimension of emotional exhaustion was the best predictor of health among health care professionals for both males and females, accounting for more than 33% of the variability in self-reported health scores. The two other dimensions of burnout, cynicism and a lack of accomplishment, taken together, accounted for only 10% of the variability in the self-reported health scores.

Third, the aforementioned indirect effect of social support on the effects of burnout exhaustion varied significantly across different indicators of health, being higher for somatic and anxiety symptomatology than for social disfunction or depression.

Fourth, effective interventions among health care professionals in Ecuador that aim to reduce burnout and related health problems may benefit from increasing perceived social support; reducing sources of stress due to conflicting roles, ambiguity, or effect–reward imbalances in the work environment; and enhancing stress copying skills by increasing control, particularly for those with higher levels of neuroticism.

## Data Availability Statement

The raw data supporting the conclusions of this article will be made available by the authors, without undue reservation.

## Ethics Statement

The studies involving human participants were reviewed and approved by the University of Loja (Ecuador). The patients/participants provided their written informed consent to participate in this study.

## Author Contributions

MR, SV and BP-C: conceptualization. PR: formal analysis and data curation, and writing – review, editing, and supervision. MR, BP-C, SV, VC-S, and PG: writing – original draft preparation. MR and PG: project administration. All authors have read and agreed to the published version of the manuscript.

## Conflict of Interest

The authors declare that the research was conducted in the absence of any commercial or financial relationships that could be construed as a potential conflict of interest.
