# Supplementary figures and images for: Social Support Mediates the Effect of Burnout on Health in Health Care Professionals
Source: Front Psychol. 2021 Jan 13;11:623587. doi: 10.3389/fpsyg.2020.623587 (PMC7838123; doi:10.3389/fpsyg.2020.623587)

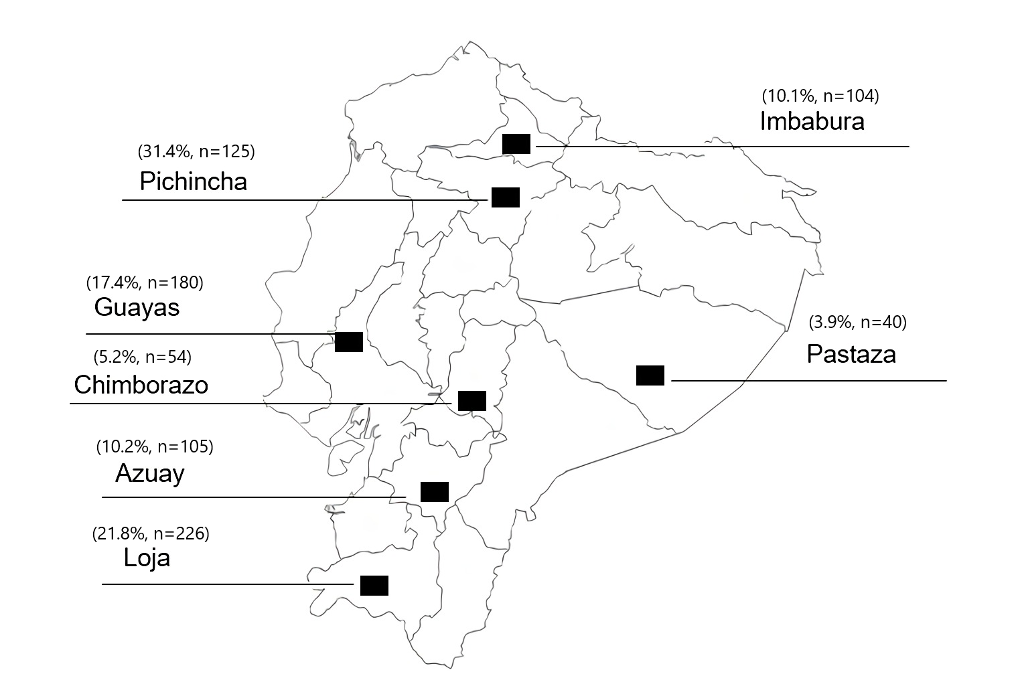

Supplement: Supplementary file 1 [file Image_1.PNG]
